# Macronutrients quality indices and risk of metabolic syndrome and its components in Iranian adults

**DOI:** 10.1186/s12872-024-03779-1

**Published:** 2024-02-26

**Authors:** Hossein Farhadnejad, Ebrahim Mokhtari, Farshad Teymoori, Mitra Kazemi Jahromi, Niloufar Saber, Hamid Ahmadirad, Mostafa Norouzzadeh, Parvin Mirmiran, Fereidoun Azizi

**Affiliations:** 1 Nutrition and Endocrine Research Center, Research Institute for Endocrine Sciences, Shahid Beheshti University of Medical Sciences, Tehran, Iran; 2 Department of Nutrition, School of Public Health, Iran University of Medical Sciences, Tehran, Iran; 3 Endocrinology and Metabolism Research Center, Hormozgan University of Medical Sciences, Bandar Abbas, Iran; 4 Department of Clinical Nutrition and Dietetics, Faculty of Nutrition Sciences and Food Technology, National Nutrition and Food Technology Research Institute, Shahid Beheshti University of Medical Sciences, Tehran, Iran; 5 Endocrine Research Center, Research Institute for Endocrine Sciences, Shahid Beheshti University of Medical Sciences, Tehran, Iran

**Keywords:** Macronutrients, Carbohydrate quality, Protein score, Metabolic syndrome, Cardiovascular risk factors, Adults

## Abstract

**Background/aim:**

Evidence from recent studies suggested that the quality of dietary macronutrients can play a possible role in predicting the risk of metabolic disorders. In the current study, we aimed to assess the association of carbohydrate quality index (CQI) and protein score with the risk of metabolic syndrome (MetS) in Iranian adults.

**Methods:**

This prospective study was conducted within the framework of the Tehran Lipid and Glucose Study on 1738 individuals aged between 40 and 70 years old, who were followed up for a mean of 6.1 years. A food frequency questionnaire was used to determine CQI and protein scores. The multivariable adjusted Cox regression model was used to calculate the hazard ratio (HR) of MetS across quartiles of protein score and CQI, and its components.

**Results:**

The mean ± standard deviation (SD) age and body mass index of the study population (42.5% men) were 49.3 ± 7.5 years and 27.0 ± 4.0 kg/m^2^, respectively. Mean ± SD scores of CQI and protein for all participants were 12.6 ± 2.4 and 10.3 ± 3.5, respectively. During the study follow-up, 834(48.0%) new cases of MetS were ascertained. In the multivariable-adjusted model, the risk of MetS was decreased across quartiles of CQI (HR = 0.83;95%CI:0.69–1.00, P_trend_=0.025) and protein score (HR = 0.75; 95% CI:0.60–0.94, P_trend_=0.041). Also, Of CQI components, the whole grain/total grains ratio showed a significant inverse association with the risk of MetS (HR = 0.75;95%CI:0.60–0.94, P_trend_=0.012).

**Conclusion:**

Our findings revealed that a dietary pattern with higher CQI and protein score may be related to a reduced risk of MetS in adults.

**Supplementary Information:**

The online version contains supplementary material available at 10.1186/s12872-024-03779-1.

## Background

Metabolic syndrome (MetS) is a collection of metabolic abnormalities that include abdominal obesity, hyperglycemia, hypertension, and dyslipidemia [1, 2]. These abnormal metabolic conditions increase the risk of developing type 2 diabetes, cardiovascular diseases (CVDs), and other chronic illnesses [3, 4], which, in turn, can contribute to disability, decreased quality of life and longevity, increased healthcare expenditure, reduced overall productivity, and slowing of economic growth and development, resulting in a reduction of gross domestic product (GDP) [5–8]. The prevalence of MetS has been increasing worldwide, with an estimated 20–25% of the global population affected [9]. A recent study found that 40% of Iranian adults are affected by MetS [10]. Given the above-mentioned significant health and economic burden of MetS, identifying modifiable factors such as diet may be effective for the prevention and management of MetS.

Dietary factors, including the quality and quantity of macronutrients, such as carbohydrates (CHO) and protein, have been identified as important contributors to the development and progression of MetS [11, 12]. Although some studies have reported an increased risk of MetS associated with higher total protein intake [13], other studies suggest that high-protein diets may have a protective effect against MetS [14]. Also, regarding the source of protein, a higher intake of animal protein was associated with an increased risk of MetS, while a higher intake of plant protein was associated with a lower risk [14]. Recently, a novel protein score has been introduced to assess the quality and quantity of dietary protein intake, which combines both total protein and the plant-to-animal ratio (PAR) [15]. Although some studies may have shown the possible role of protein score in predicting the risk of chronic diseases [16], no study has yet examined the association between the dietary protein score and the risk of MetS.

Similarly, several studies have investigated the association between CHO intake and the risk of MetS, which indicates mixed findings [17, 18]. Liu et al. showed that CHO intake is associated with an increased MetS risk [17]; however, Park et al., showed that CHO consumption was not related to MetS incidence [18]. Furthermore, in a study on elderly women, a lower intake of protein and/or higher consumption of CHOs has been associated with an increased risk of MetS [19]. Evidence suggests that the dietary CHOs’ quality may play an equally or more significant role in determining human health outcomes than the quantity of CHOs, therefore dietary recommendations have been promoting the consumption of high-quality CHOs to improve overall health and reduce the risk of chronic diseases [20].

Recently, the CHO quality index (CQI) as a scoring system that incorporates four attributes of CHO quality, namely dietary fiber, glycemic index (GI), whole grain/total grain ratio, and solid/total CHO (SCHO/TCHO) has been introduced to determine the quality and quantity of CHO in the diet [21]. Previous research has yielded interesting and valuable results and reported the possible relationship of a diet with high CQI with the reduced risk of chronic diseases, such as obesity [22, 23], CVD [24, 25], and chronic kidney disease (CKD) [16]. However, evidence on the possible association of CQI with the risk of MetS is limited to some cross-sectional studies that showed conflicting results [26, 27]. An investigation conducted on diabetic subjects suggested that a higher CQI score was inversely associated with MetS risk [27], whereas another study did not observe a significant association between CQI and odds of MetS [26]. Considering the increasing prevalence of MetS in the Iranian population and the controversy in limited current evidence on the relationship between CQI and protein score with the risk of these disorders, this study, as a prospective cohort study, aimed to investigate the association between protein score and CQI with the risk of MetS in Iranian adults.

## Materials and methods

### Study population

The present study was conducted in the framework of the Tehran lipid and glucose study (TLGS), which its protocol is comprehensively described elsewhere [28, 29]. This is an ongoing population-based study that has been run since March 1999 and aimed to identify non-communicable disease risk factors leading to better lifestyles. The first phase was conducted cross-sectionally (1999–2001) and up to now, 6 phases of TLGS have been completed. The baseline population consists of more than 15,000 people aged ≥ 3 years old. Data collection has been done every three years, and as a result, phases 2 to 6 have been carried out in 2002–2005, 2006–2008, 2009–2011, 2012–2015, and 2015–2018, respectively.

Since the collection of dietary data started from phase 3, in the present study, phases 3 and 4 are considered the baseline phases, and participants were followed until the end of phase 6. In the third survey (2006–2008), which included 12,523 people, 3568 individuals were randomly selected for dietary assessment. Meanwhile, 7956 randomly selected individuals in the fourth survey (2009–2011) agreed to complete the dietary assessment. For the current study, participants with complete dietary data on the third examination of TLGS and the new entries participants in the fourth examination who aged between 40 and 70 years were included (*n* = 3421). Among them 1819 participants were excluded due to the following reasons: (1) participants with CVD accident or myocardial infraction (*n* = 63), (2) prevalent cancer (*n* = 13), (3) pregnant and lactating women (*n* = 9), (4) those with under- or over-reported dietary energy intakes (out of the range 800–4200 kcal/day) [30] (*n* = 148), (5) participants with missing data on MetS (*n* = 65) or having MetS at the baseline (*n* = 1454) and (6) lost to follow up (*n* = 67). Of note, some of them may fall into more than one category which makes the final population equal to 1738 participants (follow-up rate: 96.2%).

### Measurements

The approach of the current study, including the measurements of demographic, clinical, anthropometric, biochemical, nutritional, and physical activity data has been explained in detail in the previous study [29]. A pretested questionnaire was used for the collection of demographic information, including age, sex, smoking status, educational level, medical history, etc. Weight, height, and waist circumference (WC) as the anthropometric indicators of the present study were measured by trained personnel using standard instruments with the highest accuracy [29]. Body mass index (BMI) was computed by dividing weight in kilograms into squares of height in meters. The systolic blood pressure (SBP) and diastolic blood pressure (DBP) were measured using a mercury sphygmomanometer and Korotkoff sound technique in a sitting position and rested for 15 min twice on the right arm (minimum interval of 30 s). The average of these two measurements was recorded as the participant’s blood pressure (BP) [29].

The physical activity levels of participants were determined using an updated and validated modifiable Activity Questionnaire (MAQ) for Iranians [31], which was reported as metabolic equivalent hours per week (MET-h/week) [29].

As described in the previous study [29], to determine biochemical variables, blood samples were collected after 12 to 14 h of fasting at the TLGS research laboratory in a sitting position and centrifuged within 30 to 45 min. Serum triglyceride (TGs), total cholesterol (TC), high-density lipoprotein cholesterol (HDL-C), and fasting plasma glucose (FPG) were measured by the enzymatic method. We did the analyses using commercial kits (Pars Azmoon Inc., Tehran, Iran) and a Selectra 2 auto-analyzer (Vital Scientific, Spankeren, Netherlands).

A validated and reliable semi-quantitative 168-item food frequency questionnaire (FFQ) was used for the collection of dietary data of participants [32] by skilled dieticians in a face-to-face manner. Dietary intake assessment has already been explained in detail [29], but briefly, we should state that. Portion sizes of consumed foods, reported in household measures were then converted to grams. Using the United States Department of Agriculture (USDA) food composition table (FCT) [33], energy and nutrient content were computed. For local food items that were not available in USDA FCT, the Iranian FCT was used [34].

The CQI [21] was calculated by summing four components, including dietary fiber intake (g/d); glycemic index (GI); whole grains: total grains ratio; and solid CHO: total CHO ratio. In the last component, only the amount of CHO from each food was considered. To compute total grains, we summed up dietary intakes of refined grains, whole grains, and their products. Liquid CHOs were defined by summing up fruit juice and sugar-sweetened beverage consumption and solid CHOs included CHOs from all other food sources [21].

The GI refers to the area under the blood glucose response curve, 2 h after consuming a food containing CHOs, compared to the same amount of CHOs from glucose. We used the international table of GI and list of the GI for Iranian foods [35, 36] to obtain the GI value of each food item, and calculate dietary GI as the following equation:

$$\begin{aligned}& {\text{Dietary GI}} \\ & \;\; = \frac{{carbohydrate\,content\,of\,each\,food\, \times \,number\,of\,servings/day\,}}{{Total\,daily\,carbohydrate\,intake}} \\ & \;\;\;\;\; \times {\text{GI}} \\ \end{aligned}$$

In all components, the individuals were classified into quintiles according to the intake of the above components and then were assigned a value that ranged from 1 to 5. For the glycemic index, the participants who were in the first quintile received 5 points, and those in the fifth one were assigned 1 point. For other components, the individuals who were in the highest quintile were given 5 scores and those in the lowest one received 1 score. To compute the CQI score that varied from 4 to 20, the calculated score for four components was summed. Also, the score of each component was considered and reported separately [21].

The protein score [15] is based on two bases, including the percentage of protein from the total energy intake and the ratio of PAR. In this context, the population was divided into 11 equal categories based on the score they got from each item (PAR and percent of protein from energy). Meanwhile, the lowest to highest category gets a score between 0 and 10, respectively. Finally, with the sum of the scores of these two items, people get a score between 0 and 20, which is the higher score indicating a higher relative consumption of protein or higher PAR. For this purpose, the index components are also reported separately [16].

### Definitions

#### Metabolic syndrome (MetS)

We defined MetS based on the joint interim statement as the existence of three or more of the following factors [29, 37]: (1) FPG ≥ 100 mg/dl or previously diagnosed type 2 diabetes, (2) TG ≥ 150 mg/dl or specific treatment for dyslipidemia (fibric acid derivatives, statins, resins, niacin, omega-3 fatty acids, and their combinations), (3) HDL-C < 40 mg/dl (males) or < 50 mg/dl (females) or drug treatment (niacin, fibrates, and certain statins), (4) SBP ≥ 130 mmHg or DBP ≥ 85 mmHg or treatment of previously diagnosed hypertension, and (5) WC ≥ 95 cm for both genders [38].

### Statistical analyses

Statistical analysis was conducted using the Statistical Package for the Social Sciences (SPSS) software (version 15.0, SPSS Inc., Chicago, IL, USA). The normality of the variables was checked using histogram charts and Kolmogorov–Smirnov test. Baseline characteristics of participants are reported as mean ± standard deviation (SD) or median (25–75 interquartile range) for quantitative variables and percentages for categorical variables in participants. The independent two-sample t-test was used to compare the mean values of quantitative variables between MetS and non-MetS groups. Also, the chi-square test was used to compare the categorical variables between MetS and non-MetS groups.

The relationship between protein score, CQI, and their components with changes in MetS factors including WC, TG, HDL-C, FPG, SBP, and DBP was determined using a linear regression test in the total population, individuals with MetS, and non-MetS subjects. The final linear regression model was adjusted for age, sex, smoking (yes, no), physical activity, energy intake, and baseline values of every MetS-dependent component. For each component of MetS, unstandardized β [95% CI (confidence interval)] and p-value were reported.

Individuals’ person-time (person-year) and duration of follow-up (in the year) were computed from baseline to the time at which an event (definitive diagnosis of MetS according to the above-mentioned definition) occurred for the first time (event date), or the last date of follow-up examination, whichever occurred first. The event date of occurrence of the MetS was considered as mid-time between the date of the follow-up visit at which the MetS was identified for the first time and the most recent follow-up visit preceding the diagnosis. Cox proportional hazard regression was used to determine the risk of MetS incidents across quartiles of protein score, CQI, and their components. We reported the hazard ratios (HRs) 95%CI according to two models, including model 1 (adjusted for age, sex, smoking, physical activity, and energy intake) and model 2 (adjusted for Model 1 and baseline values of SBP, FPG, TG to HDL-C ratio, WC). All *P*-values were based on two-sided tests, and *P*-values *<* 0.05 were considered significant.

## Results

The mean ± SD age and BMI of the study population (42.5% men) were 49.3 ± 7.5 years and 27.0 ± 4.0 kg/m^2^, respectively. Mean ± SD scores of CQI and protein for all participants were 12.6 ± 2.4 and 10.3 ± 3.5, respectively. During the study follow-up, 834(48.0%) new cases of MetS were ascertained.

Table 1 indicates the data on general characteristics, biochemical measurements, and dietary intakes of participants in the MetS and MetS groups. Participants with MetS were more likely to be older, female, and have a higher mean of SBP, FPG, TGs, waist, and TG to HDL-C ratio compared to non-MetS individuals. However, HDL-C was significantly higher in the healthy group compared to participants in the MetS group. There was no significant difference in physical activity level, smoking, and dietary intake between participants in the MetS and non-MetS groups.

**Table 1:** Baseline characteristics of participants according to metabolic syndrome incidence

Variables	Healthy(*n* = 904)	Metabolic syndrome patients (*n* = 834)	P-value*
Age (years)	48.8 ± 7.5	49.7 ± 7.4	0.017
Male (%)	45.1	39.6	0.019
Smoking (%)	15.5	12.5	0.066
Physical activity (MET/hour/week)	53.5 (10.4–99.0)	54.3 (11.9–96.3)	0.611
SBP (mmHg)	109.8 ± 14.4	113.6 ± 14.6	< 0.001
FPG (mg/dl)	88.6 ± 13.1	93.2 ± 24.5	< 0.001
TGs (mg/dl)	115.4 ± 60.3	149.9 ± 75.5	< 0.001
HDL-c(mg/dl)	46.6 ± 10.7	42.3 ± 9.7	< 0.001
TG to HDL-c ratio	2.70 ± 1.85	3.87 ± 2.78	< 0.001
Waist(cm)	87.3 ± 9.8	92.4 ± 9.0	< 0.001
**Dietary intakes**			
Energy(Kcal/d)	2250 ± 684	2233 ± 686	0.609
Carbohydrate(% of energy)	56.7 ± 6.3	56.8 ± 6.4	0.639
Fat(% of energy)	29.2 ± 6.4	29.2 ± 6.5	0.897
Protein score	10.5 ± 3.5	10.2 ± 3.4	0.125
Protein(% of energy)	14.0 ± 2.7	13.9 ± 2.8	0.434
Plant protein(% of energy)	7.56 ± 1.74	7.45 ± 1.63	0.186
Animal protein(% of energy)	6.92 ± 2.62	7.00 ± 2.66	0.532
PAR	1.33 ± 0.92	1.30 ± 1.03	0.483
CQI	12.6 ± 2.3	12.5 ± 2.5	0.229
Fiber (g/1000Kcal)	19.6 ± 8.4	19.2 ± 6.6	0.281
Glycemic index(1000/Kcal)	28.6 ± 10.6	28.7 ± 10.9	0.727
whole-grain/total-grain	0.27 (0.13–0.43)	0.26 (0.12–0.41)	0.332
SCHO /TCHO	0.98 ± 0.02	0.98 ± 0.02	0.776

*Abbreviations* SBP: Systolic blood pressure, FPG: fasting plasma glucose, TGs: Triglycerides, PAR: Plant: animal protein ratio, CQI: carbohydrates quality index, SCHO: solid carbohydrates, TCHO: Total carbohydrates (including solid carbohydrates + liquid carbohydrates)

Data represented as mean ± SD, or median (IQR 25–75) for continuous variables and percent for categorical variables

* Independent t-test and chi-square were used to test the differences between groups for continuous and categorical variables, respectively

TGs and HDL-C change per increment one Z score of CQI, protein score, and their components in the total population, healthy individuals, and MetS groups during the 6-year follow-up of the study. There is no significant association between CQI and protein score with 6-year TGs change. However, the SCHO/TCHO ratio as an important CQI component in the total population (β =-8.45; 95% CI: -15.7– -1.14, P for trend = 0.023) and the MetS group (β =-14.1; 95% CI: -25.3– -2.9, P for trend = 0.013) were inversely associated with 6-year TGs changes. Also, PAR in the MetS group (β=-5.17; 95% CI: -10.2– -0.05, P for trend = 0.048) was inversely associated with 6-year TG changes. However, there is no significant association between their other CQI, and protein score components with TGs change.

Table 2 showed that no significant association was found between CQI and 6-change in HDL-C level in all study groups. However, among its components, higher fiber intake (β = 1.65; 95% CI: 0.32– 2.98, *P* = 0.015) was related to increasing HDL-C level during the 6-year follow-up in the MetS group. Protein score (β = 0.44; 95% CI: 0.04– 0.83, *P* = 0.028) and plant protein (β = 0.43; 95% CI: 0.02– 0.83, P for trend = 0.039) in the total population was positively related to 6- years change in HDL-C level. Also, PAR (β = 0.76; 95% CI: 0.07– 1.45, *P* = 0.030) in the MetS group, and total protein (β = 1.00; 95% CI: 0.42– 1.58, *P* = 0.001), and animal protein (β = 0.79; 95% CI: 0.22– 1.37, *P* = 0.007) in the healthy group have a positive significant association with 6-year HDL-C change.

**Table 2:** Beta regression (95% CI) of 6-year changes of triglycerides and high density lipoprotein-cholesterol per increment of each Z score of dietary carbohydrate and protein indices

Dietary indices*	Total population(*n* = 1738)		Healthy(*n* = 904)		Metabolic syndrome patients (*n* = 834)	
	**B (95% CI**)	**P-value**	**B (95% CI**)	**P-value**	**B (95% CI**)	**P-value**
**TGs changes**						
CQI	-0.99 (-3.62–1.63)	0.458	1.57 (-1.49–4.63)	0.315	-2.29(-6.40–1.81)	0.273
*GI*(per 1000Kcal)	-3.08 (-8.27– 2.10)	0.243	-0.91 (-7.10 − 5.27)	0.772	-5.12(-13.0–2.79)	0.204
*Fiber intake*(g/1000Kcal)	-3.22 (-9.35–2.89)	0.301	1.13 (-5.76 − 8.03)	0.746	-5.12(-15.0–4.77)	0.310
*Whole grain/total grains*	-1.82 (-4.59–0.94)	0.196	1.28 (-1.93–4.49)	0.435	-4.00(-8.34–0.33)	0.070
*SCHO /TCHO*	-8.45 (-15.7– -1.14)	0.023	-2.26 (-11.0 − 6.46)	0.611	-14.1(-25.3– -2.9)	0.013
Protein score	-0.89 (-3.44–1.64)	0.48	2.27 (-0.58 − 5.13)	0.119	-3.08(-7.19–1.01)	0.140
*Total protein (% of energy)*	0.44 (-2.29 − 3.18)	0.75	1.73 (-1.40 − 4.87)	0.278	-0.50(-4.86–3.85)	0.820
*Plant protein*	-1.01 (-3.65–1.63)	0.45	2.16 (-0.75– 5.08)	0.146	-3.47(-7.82–0.88)	0.118
*Animal protein*	1.21 (-1.52–3.95)	0.38	0.00 (-3.11–3.13)	0.996	1.94 (-2.44–6.33)	0.384
*PAR*	-2.58 (-5.60 − 0.43)	0.09	0.51 (-2.79–3.81)	0.761	-5.17(-10.2–-0.05)	0.048
**HDL-c changes**						
CQI	0.33 (-0.07 − 0.74)	0.106	0.04 (-0.52–0.61)	0.868	0.53 (-0.01–1.09)	0.058
*GI*	-0.39 (-1.19–0.40)	0.337	-0.52 (-1.67– 0.62)	0.367	-0.38(-1.45–0.67)	0.475
*Fiber intake*	0.67 (-0.27– 1.61)	0.163	-0.45 (-1.73– 0.82)	0.485	1.65 (0.32–2.98)	0.015
*Whole grain/total grains*	0.24 (-0.18–0.67)	0.264	0.07 (-0.52– 0.67)	0.816	0.34 (-0.24–0.92)	0.255
*SCHO /TCHO*	-0.14 (-1.27– 0.99)	0.805	-0.21 (-1.83–1.41)	0.798	0.01 (-1.49–1.53)	0.983
Protein score	0.44 (0.04–0.83)	0.028	0.32 (-0.20 − 0.85)	0.228	0.45 (-0.10–1.01)	0.109
*Total protein (% of energy)*	0.40 (-0.01– 0.83)	0.058	1.00 (0.42 − 1.58)	0.001	-0.14(-0.72–0.44)	0.641
*Plant protein*	0.43 (0.02–0.83)	0.039	0.20 (-0.34–0.74)	0.466	0.58 (-0.00–1.17)	0.050
*Animal protein*	0.09 (-0.33–0.51)	0.674	0.79 (0.22–1.37)	0.007	-0.50(-1.09–0.08)	0.094
*PAR*	0.11 (-0.34–0.58)	0.623	-0.56 (-1.17–0.05)	0.073	0.76(0.07–1.45)	0.030

*Abbreviations* TGs: triglycerides, HDL-C: high-density lipoprotein cholesterol, CQI: carbohydrate quality index, PAR: Plant-to-animal protein ratio, SCHO /TCHO: solid carbohydrates/ total carbohydrates, GI: Glycemic index

Analyses were adjusted for age and sex, smoking (yes, no), physical activity, energy intake, and baseline values for each dependent metabolic syndrome component

Table 3 reported 6-y SBP and DBP change per one Z score of CQI, protein score, and their components in the total population, healthy individuals, and MetS groups. There was no significant relationship between CQI and 6-y SBP and DBP change in the total population, MetS, and non-MetS groups. However, high dietary GI in the total population (β = 1.60; 95% CI: 0.17– 3.03, *P* = 0.028) and MetS group (β = 2.34; 95%CI: 0.36– 4.33, *P* = 0.021) associated with increased SBP level during 6 years follow-up. Also, SCHO/TCHO ratio in the total population (β = 2.87; 95% CI: 0.85– 4.89, *P* = 0.005) and non-MetS group (β = 4.03; 95% CI: 1.24–6.82, *P* = 0.005) had a positive significant relationship with the 6-year change of SBP mean.

**Table 3:** Beta regression (95% CI) of 6-year changes of systolic blood pressure and diastolic blood pressure per increment of each Z score of dietary carbohydrate and protein indices

Dietary indices*	Total population(*n* = 1738)		Healthy(*n* = 904)		Metabolic syndrome patients (*n* = 834)	
	**B (95% CI**)	**P-value**	**B (95% CI**)	**P-value**	**B (95% CI**)	**P-value**
**SBP changes**						
CQI	-0.32 (-1.05–0.40)	0.378	0.54 (-0.44 − 1.52)	0.281	-0.94(-1.97–0.08)	0.073
*GI*(per 1000Kcal)	1.60 (0.17–3.03)	0.028	1.03 (-0.94–3.02)	0.305	2.34 (0.36–4.33)	0.021
*Fiber intake*(g/1000Kcal)	0.66 (-1.03–2.37)	0.444	1.20 (-1.00–3.41)	0.283	0.33 (-2.19–2.86)	0.796
*Whole grain/total grains*	-0.52 (-1.28–0.24)	0.128	0.15 (-0.87–1.18)	0.771	-0.97(-2.06–0.11)	0.079
*SCHO /TCHO*	2.87 (0.85–4.89)	0.005	4.03 (1.24–6.82)	0.005	2.01(-0.80 − 4.82)	0.161
Protein score	-0.73 (-1.43– -0.02)	0.042	-0.98(-1.89–-0.06)	0.000	-0.33(-1.36–0.69)	0.520
*Total protein (% of energy)*	-0.85 (-1.6 – -0.10)	0.026	-1.34(-2.35–-0.34)	0.008	-0.50(-1.59–0.59)	0.369
*Plant protein*	-0.51 (-1.24–0.21)	0.165	-0.46(-1.39– 0.47)	0.336	-0.42(-1.52–0.66)	0.443
*Animal protein*	-0.42 (-1.18–0.32)	0.265	-0.92(-1.91–0.07)	0.069	-0.18(-1.28–0.91)	0.740
*PAR*	-0.05 (-0.89–0.78)	0.900	0.54 (-0.51– 1.60)	0.316	-0.28(-1.57–1.01)	0.669
**DBP changes**						
CQI	0.07 (-0.34–0.50)	0.726	0.42(-0.16–1.01)	0.154	-0.12(-0.72–0.47)	0.674
*GI*	-0.13 (-0.97–0.69)	0.744	-0.21(-1.39–0.96)	0.724	-0.01(-1.16–1.13)	0.980
*Fiber intake*	-0.45 (-1.44–0.54)	0.374	-0.05(-1.36–1.26)	0.939	-0.67(-2.14–0.78)	0.362
*Whole grain/total grains*	-0.07 (-0.51–0.37)	0.752	0.27(-0.34–0.88)	0.387	-0.27(-0.90–0.35)	0.395
*SCHO /TCHO*	-0.00 (-1.18–1.17)	0.993	1.17(-0.48–2.84)	0.165	-0.98(-2.61–0.64)	0.237
Protein score	-0.46 (-0.87– -0.05)	0.027	-0.41(-0.96–0.13)	0.138	-0.40(-1.00–0.18)	0.180
*Total protein (% of energy)*	-0.37 (-0.81–0.06)	0.095	-0.57(-1.17–0.01)	0.057	-0.20(-0.83–0.43)	0.535
*Plant protein*	-0.47 (-0.89– -0.04)	0.029	-0.37(-0.93–0.18)	0.189	-0.47(-1.11–0.15)	0.139
*Animal protein*	-0.02 (-0.46–0.41)	0.911	-0.27(-0.86–0.31)	0.363	0.12(-0.51–0.76)	0.706
*PAR*	-0.31 (-0.80–0.17)	0.202	-0.13(-0.76–0.50)	0.687	-0.30(-1.05–0.43)	0.419

*Abbreviations* SBP: systolic blood pressure, DBP: diastolic blood pressure, CQI: carbohydrate quality index, PAR: Plant-to-animal protein ratio, SCHO /TCHO: solid carbohydrates/ total carbohydrates, GI: glycemic index

Analyses were adjusted for age and sex, smoking (yes, no), physical activity, energy intake, and baseline values for each dependent metabolic syndrome component

Table 3 indicated that high protein score in the total population [(β=-0.73; (95%CI:-1.43, -0.02), *P* = 0.042)] and non-MetS group [(β= -0.98; (95% CI:-1.89, 0.06), *P* < 0.001)] was inversely associated with 6-year SBP change. Also, a high protein score was related to a decrease in the 6-y change in DBP level in the total population [(β=-0.46; (95%CI: -0.87, -0.05), *P* = 0.027)]. Of protein score components, total protein in the total population [(β=-0.85; (95%CI:-1.60, -0.10), *P* = 0.026)] and non-MetS group [(β=-1.34; (95%CI:-2.35, -0.34), *P* = 0.008)] were inversely associated with SBP level change. Also, a negative association was observed between plant protein [(β=-0.47; (95%CI: -0.89, -0.04), *P* = 0.029)] with the 6-year change of DBP in the total population. Other components of protein score and CQI had no significant relationship with 6-year changes in SBP and DBP levels in any of the three above-mentioned groups.

The association between CQI, protein score, and their components with change in FPG and WC levels during the 6-year follow-up of the study was shown in Table 4. There is no significant association between CQI, protein score, and their components with a 6-y change in FPG mean. For the 6-y WC changes, only total protein intake in the MetS group [(β=-0.54; (95% CI: -1.09, 0.00, *P* = 0.048)] showed an inverse association. However, no significant relationship was observed between protein score and its other components with a 6-y change in WC level in all three groups.

**Table 4:** Beta regression (95% CI) of 6-year changes of fasting plasma glucose and waist circumference per increment each Z score of dietary carbohydrate and protein indices

Dietary indices*	Total population(*n* = 1738)		Healthy(*n* = 904)		Metabolic syndrome patients (*n* = 834)	
	**B (95% CI**)	**P-value**	**B (95% CI**)	**P-value**	**B (95% CI**)	**P-value**
**FPG changes**						
CQI	-0.31(-1.15– 0.52)	0.466	0.14(-0.64–0.93)	0.716	-0.49(-1.92–0.92)	0.492
*GI*(per 1000Kcal)	-0.92(-2.58– 0.74)	0.278	-0.90(-2.49–0.68)	0.262	-0.92(-3.66–1.82)	0.511
*Fiber intake*(g/1000Kcal)	-0.17(-2.17–1.78)	0.862	0.06(-1.69–1.82)	0.943	-0.05(-3.48–3.36)	0.973
*Whole grain/total grains*	0.07(-0.81– 0.95)	0.872	0.32(-0.49–1.15)	0.433	0.20(-1.30–1.70)	0.790
*SCHO /TCHO*	-0.72(-3.07–1.62)	0.544	-0.43(-2.68–1.81)	0.705	-0.68(-4.56–3.19)	0.728
Protein score	-0.07(-0.89–0.73)	0.848	0.44(-0.28–1.17)	0.229	-0.50(-1.92–0.91)	0.487
*Total protein (% of energy)*	0.53(-0.34–1.41)	0.232	0.38(-0.42–1.19)	0.349	0.39(-1.10–1.90)	0.604
*Plant protein*	0.12(-0.71–0.97)	0.764	0.47(-0.26–1.22)	0.208	-0.12(-1.62–1.38)	0.875
*Animal protein*	0.45(-0.42–1.32)	0.311	0.06(-0.73–0.86)	0.871	0.44(-1.06–1.96)	0.562
*PAR*	-0.39(-1.35–0.57)	0.427	-0.10(-0.95–0.73)	0.799	-0.12(-1.89–1.64)	0.889
**Waist changes**						
CQI	-0.04(-0.40–0.31)	0.815	0.11(-0.38–0.60)	0.656	-0.10(-0.62–0.40)	0.681
*GI*	-0.11(-0.82–0.60)	0.762	-0.06(-1.07–0.93)	0.892	-0.15(-1.14–0.83)	0.759
*Fiber intake*	0.38(-0.45–1.22)	0.366	0.17(-0.93–1.28)	0.756	0.70(-0.53–1.94)	0.264
*Whole grain/total grains*	-0.22(-0.60–0.15)	0.244	0.05(-0.46–0.57)	0.835	-0.33(-0.87–0.21)	0.220
*SCHO /TCHO*	-0.60(-1.60–0.39)	0.237	-0.30(-1.72–1.11)	0.673	-0.82(-2.21–0.55)	0.241
Protein score	-0.06(-0.41–0.28)	0.726	0.13(-0.32–0.59)	0.567	-0.20(-0.72–0.30)	0.431
*Total protein (% of energy)*	-0.25(-0.63–0.11)	0.178	0.03(-0.47–0.54)	0.887	-0.54(-1.09–0.00)	0.048
*Plant protein*	-0.06(-0.43–0.29)	0.710	0.04(-0.42–0.51)	0.839	-0.13(-0.67–0.41)	0.630
*Animal protein*	-0.17(-0.55–0.19)	0.347	0.02(-0.47–0.52)	0.924	-0.44(-0.98–0.10)	0.112
*PAR*	0.06(-0.35–0.48)	0.763	-0.04(-0.58–0.49)	0.864	0.30(-0.33–0.94)	0.348

*Abbreviations* FPG: fasting plasma glucose, CQI: carbohydrate quality index, PAR: Plant-to-animal protein ratio, SCHO /TCHO: solid carbohydrates/ total carbohydrates, GI: Glycemic index

Analyses were adjusted for age and sex, smoking (yes, no), physical activity, energy intake, and baseline values for each dependent metabolic syndrome component

Table 5 indicates the association of CQI and its components, including dietary GI, fiber intake, whole grain/total grains ratio, and SCHO/TCHO ratio with MetS incident. In model 1, after controlling the effects of age, sex, smoking, physical activity, and energy intake, the association between the above-mentioned indices and the incidence of MetS was non-significant. However, in the final model, after adjusting for the potential confounders, including age, sex, smoking, physical activity, energy intake, baseline values of SBP, FPG, triglycerides to HDL-C ratio, and WC, the risk of MetS incident in individuals in the highest quartile of CQI (HR = 0.83; 95% CI: 0.69–1.00, P for trend = 0.025) and whole grain/total grains ratio (HR = 0.75; 95% CI: 0.60– 0.94, P for trend = 0.012) was significantly lower than those in those in the first quartile. Based on the multivariable model, there was no significant association between other CQI components and the risk of MetS.

**Table 5:** Hazard ratio (95% CI) of metabolic syndrome incidence across carbohydrate quality index score and its components

	Carbohydrate quality index and its components						
**Dietary indices**	Q1	Q2	Q3	Q4	P-trend	Per 1 SD	P-value
**Carbohydrate quality index**							
Median score	10.0	12.0	14.0	16.0			
Follow up period	6.1	6.2	6.9	6.8			
Model 1*	1.00 (Ref)	0.97 (0.82–1.16)	0.84 (0.67–1.05)	0.87 (0.72–1.06)	0.100	0.93 (0.87–1.00)	0.065
Model 2^†^	1.00 (Ref)	0.98 (0.82–1.17)	0.81 (0.64–1.01)	0.83 (0.69–1.00)	0.025	0.92 (0.85–0.98)	0.023
***Glycemic index*** (per 1000Kcal)							
Median score	18.0	23.6	29.9	41.1			
Follow up period	6.5	6.8	6.8	6.9			
Model 1*	1.00 (Ref)	1.09 (0.8–1.38)	1.09 (0.82–1.45)	0.98 (0.68–1.42)	0.571	1.00 (0.87–1.15)	0.926
Model 2^†^	1.00 (Ref)	1.12 (0.88–1.41)	1.13 (0.84–1.51)	1.11 (0.76–1.61)	0.871	1.01 (0.89–1.16)	0.792
Model 3^‡^	1.00 (Ref)	1.02 (0.80–1.31)	1.01 (0.73–1.36)	0.92 (0.61–1.38)	0.493	0.94 (0.81–1.10)	0.480
***Fiber intake***(g/1000Kcal)							
Median score	12.5	16.6	20.2	26.7			
Follow up period	6.3	7.0	6.4	7.0			
Model 1*	1.00 (Ref)	0.91 (0.75–1.11)	0.92 (0.76–1.12)	0.91 (0.75–1.10)	0.309	0.89 (0.75–1.06)	0.209
Model 2^†^	1.00 (Ref)	0.90 (0.74–1.09)	1.02 (0.84–1.24)	0.90 (0.74 − 1.09)	0.481	0.90 (0.76–1.07)	0.262
Model 3^‡^	1.00 (Ref)	0.93 (0.76–1.13)	1.05 (0.86–1.29)	0.91 (0.75–1.11)	0.996	0.91 (0.77–1.08)	0.304
***Whole grain/total grains***							
Median score	0.03	0.08	0.15	0.26			
Follow up period	6.6	6.4	6.8	6.7			
Model 1*	1.00 (Ref)	0.99 (0.81–1.20)	0.93 (0.77–1.13)	0.85 (0.69 − 1.04)	0.090	0.95 (0.88–1.02)	0.181
Model 2^†^	1.00 (Ref)	0.94 (0.78–1.14)	0.92 (0.76 − 1.12)	0.77 (0.63 − 0.95)	0.016	0.94 (0.85–0.99)	0.036
Model 3^‡^	1.00 (Ref)	0.92 (0.76–1.12)	0.89 (0.73–1.09)	0.75 (0.60–0.94)	0.012	0.91 (0.84–0.99)	0.033
***SCHO/TCHO***							
Median score	0.31	0.40	0.50	0.67			
Follow up period	6.6	6.4	7.1	6.2			
Model 1*	1.00 (Ref)	1.25 (0.93–1.68)	1.11 (0.74–1.66)	1.19 (0.69–2.05)	0.851	1.01 (0.83–1.23)	0.858
Model 2^†^	1.00 (Ref)	1.20 (0.89–1.61)	0.99 (0.66–1.49)	1.03 (0.60–1.79)	0.516	0.96 (0.79–1.16)	0.697
Model 3^‡^	1.00 (Ref)	1.27 (0.90–1.63)	1.02 (0.68–1.53)	1.09 (0.63–1.88)	0.642	0.98 (0.81–1.20)	0.989

*Abbreviations* SCHO/ TCHO: Solid carbohydrates/Total carbohydrates ratio

*Model 1: adjusted for age and sex, smoking (yes, no), physical activity, and energy intake

^†^ Model 2: additionally adjusted for baseline values of systolic blood pressure, fasting blood sugar, triglycerides to HDL-C ratio, and waist

^‡^ Model 3: additionally other components of the CQI index were adjusted

The HRs (95%CI) of MetS according to quartiles of protein score, and its components, including total protein, plant protein, animal protein, and PAR were presented in Table 6. In model 1, after adjusting for age, sex, smoking, physical activity, and energy intake, there was no significant relationship between protein score, total protein, plant protein, animal protein, and PAR and risk of MetS incidents. After adjustment for all potentially confounding variables in the multivariable model, we observed a negative relationship between protein score and MetS incident e (HR = 0.75; 95% CI: 0.60– 0.94, P for trend = 0.041). However, no significant association was found between protein score components and the risk of MetS based on the multivariable model.

**Table 6:** Hazard ratio (95% CI) of metabolic syndrome incidence across protein score and its components

**Dietary indices**	Protein score and its components						
	Q1	Q2	Q3	Q4	P-trend	Per 1 SD	P-value
**Protein score**							
Median score	7.0	10.0	12.0	15.0			
Follow up period	6.6	7.1	6.4	6.6			
Model 1*	1.00 (Ref)	0.90 (0.75–1.09)	1.00 (0.83–1.20)	0.84 (0.68–1.05)	0.269	0.96 (0.89–1.02)	0.255
Model 2^†^	1.00 (Ref)	0.88 (0.73–1.06)	0.97 (0.81–1.17)	0.75 (0.60–0.94)	0.041	0.93 (0.87–0.99)	0.040
***Total protein (% of energy)***							
Median score	12.0	14.6	15.6	17.9			
Follow up period	6.9	7.1	6.0	6.7			
Model 1*	1.00 (Ref)	0.87 (0.72–1.06)	1.05 (0.87–1.27)	0.84 (0.68–1.02)	0.222	0.98 (0.91 − 1.05)	0.579
Model 2^†^	1.00 (Ref)	0.94 (0.77–1.15)	1.08 (0.89–1.31)	0.82 (0.67–1.00)	0.115	0.97 (0.90 − 1.04)	0.427
***Plant protein***							
Median score	5.8	7.1	8.1	9.7			
Follow up period	6.9	6.5	6.4	6.8			
Model 1*	1.00 (Ref)	1.07 (0.88–1.30)	1.02 (0.84–1.24)	0.91 (0.75–1.11)	0.309	0.96 (0.89 − 1.03)	0.316
Model 2^†^	1.00 (Ref)	1.13 (0.93–1.37)	1.00 (0.82–1.22)	0.90 (0.74–1.11)	0.202	0.93 (0.87–1.00)	0.075
Model 3^‡^	1.00 (Ref)	1.13 (0.93–1.38)	1.01 (0.83–1.23)	0.91 (0.73–1.12)	0.243	0.93 (0.86–1.00)	0.086
***Animal protein***							
Median score	4.3	6.1	7.8	10.0			
Follow up period	7.1	6.3	6.8	6.5			
Model 1*	1.00 (Ref)	1.16 (0.96–1.41)	1.05 (0.86–1.28)	1.01 (0.82–1.23)	0.820	1.00 (0.93–1.08)	0.820
Model 2^†^	1.00 (Ref)	1.18 (0.97–1.43)	1.09 (0.89–1.33)	1.08 (0.88–1.32)	0.642	1.01 (0.94 − 1.09)	0.606
Model 3^‡^	1.00 (Ref)	1.16 (0.95–1.40)	1.05 (0.85–1.29)	1.02 (0.82–1.26)	0.882	0.99 (0.91–1.07)	0.865
***PAR***							
Median score	0.63	0.95	1.30	2.0			
Follow up period	5.8	6.7	6.6	7.0			
Model 1*	1.00 (Ref)	1.14 (0.94–1.38)	1.01 (0.83 − 1.23)	0.99 (0.81–1.21)	0.602	0.96 (0.88–1.04)	0.340
Model 2^†^	1.00 (Ref)	1.14 (0.94–1.38)	1.01 (0.83–1.24)	0.91 (0.74–1.12)	0.166	0.94 (0.86–1.03)	0.184

*Abbreviations* PAR: plant protein/ animal protein ratio

*Model 1: adjusted for age and sex, smoking (yes, no), physical activity, and energy intake

^†^ Model 2: additionally adjusted for baseline values of systolic blood pressure, fasting blood sugar, triglycerides to HDL-c ratio, and waist

^‡^ Model 3: additionally animal and plant proteins were mutually adjusted for each other

## Discussion

In the present study, we investigated the association of dietary CHO and protein quality indices and their components with the incidence of MetS in the Iranian population. In summary, the results of the current study revealed that a dietary pattern with a higher score of CQI and protein index can be inversely related to the risk of MetS. Also, the score of whole grains to total grains ratio, which is one of the components of the CQI, was associated with a reduction in the risk of MetS.

The current study’s results align with previous research on the link between CHO quantity and quality in the diet and the risk of MetS or its components, which has yielded conflicting findings [26, 27, 39, 40]. Our findings align with Suara et al.‘s study [27] showing a higher CQI score was inversely linked to MetS risk in type 2 diabetes. Another study suggested a diet with a higher CQI score may be inversely related to elevated blood pressure, an important component of MetS [39]. Another study by Majidi et al. [26] found no link between CQI and MetS and its components. Also, a study in the Health Survey of São Paulo showed that dietary GI and GL were not associated with odds of MetS [40]. The important advantage of our study compared to most of the above-mentioned studies is that these studies have a cross-sectional design and could not accurately estimate the cause-effect relationship between CQI and the risk of MetS. Also, some studies have only focused on one aspect of dietary CHO quality, such as GI or GL [40], or have only examined the relationship between dietary CQI and one component of MetS, such as high blood pressure [39], however, in the present study, we made a more comprehensive and accurate definition to determine the exposure (CQI) and the outcome (MetS) based on several components that helped us to show more specifically the relationship between CQI and the risk of MetS. Furthermore, unlike our study, which has been conducted on individuals without chronic metabolic diseases and has higher generalizability for adults in society, some previous studies had been focused on subjects with chronic diseases such as obesity [39] or diabetes [27]; these above- mentioned point could be a source of controversy in the results of studies.

Although the results of past studies on the possible association between the ratio of whole grains to total grains with MetS risk have been controversial, consistent with our findings, a recent meta-analysis by Guo et al. [38] found that eating more whole grains lowers MetS risk while eating more refined grains raises it. The possible mechanisms proposed in past studies emphasize two facts in this regard; diets containing higher amounts of whole grains naturally have lower GI and glycemic load (GL) [41]. In previous studies, it has been shown that GI and GL are directly related to the risk of MetS, which is independent of diabetes [42]. Also, diets rich in whole grains are usually nutritious and rich in fiber, vitamins, minerals, and phytochemicals that are effective in reducing the risk of MetS [43]. High intake of fiber and anti-oxidant compounds from a low-GI diet containing high whole grains can have a significant effect on increasing the level of adiponectin and decreasing interleukin-6, C-reactive protein, and tumor necrosis factor-alpha, which these metabolic and hormonal changes may improve the level of metabolic parameters such as plasma levels of TGs, total cholesterol, fatty acids, blood glucose, blood pressure [44–46], while refined grains are poor in micronutrient content and antioxidant compounds, and so their role in predicting the risk of metabolic disorders may be against whole grains [47]. Furthermore, chewing whole grains that have a high content of solid CHO may create a feeling of fullness that is not experienced when consuming refined cereals. Early oral exposure to thick or solid substances triggers pancreatic responses sooner than oral exposure to fluids [48–50]. Furthermore, liquids empty faster from the stomach and are absorbed more quickly in the intestine, leading to increased insulin release and endocrine disorders, ultimately increasing Denovo TG synthesis [51].

In our study, we did not find that the combined effect of CQI components was stronger than the individual components in predicting the risk of MetS. The association of CQI with reducing the risk of MetS was more influenced by the whole grain/total grains ratio. The similar amount received by the participants and the lack of significant variation in their consumption for other CQI components may justify these findings. In the current study population, which is a representative sample of Iranian society, the SCHO/TCHO ratio is 98%, and 94.2% of the participants in our study have an SCHO/TCHO ratio > 95%; this issue limits the possibility of separating individuals into low and high SCHO/TCHO intakes and therefore we can almost show that our population is completely homogeneous and very close to each other for this CQI component. Therefore, the effect of SCHO/TCHO on the CQI effect is weakened. Fiber intake is another component of CQI that should be considered in this case; the baseline results in the present study showed that about 80% of our population have a fiber intake > 14 g per 1000 kcal of energy intake (the recommended amount for adults) in their daily diet. In a population where over 80% have adequate or excessive fiber intake and there is little variation in fiber intake, it will not be possible to accurately study the impact of fiber on chronic disease occurrence. In our study, the impact of fiber intake on the overall predictive power of the CQI index for the risk of MetS is not expected to be significant. Dietary recommendations typically emphasize consuming foods with low (< 55) and medium (55–70) GI for better blood sugar control [52]. Our study found that only 8.5% of the participants had a diet with a GI > 70. Most of the participants had a diet with low or medium GI, indicating a relatively good condition for GI index. There was not much variation in the dietary GI among them. These findings suggest that the contribution of the GI index to the total CQI score in predicting the risk of MetS may not vary significantly among the study population. In our study, about 30% of the population had a whole grain/total grains ratio of < 0.15, 40% had a ratio between 0.15 and 0.4, and 30% had a ratio > 0.4, showing significant variation in whole grain intake. After adjusting for other factors, a higher whole grain/total grains ratio was linked to a lower risk of MetS.

Although no prospective study has looked at the relationship between protein score and the risk of MetS, our findings on the inverse association between higher protein score and MetS risk align with a previous study on Iranian adults. This study showed that a diet with a high protein score, characterized by a higher intake of plant proteins and a lower intake of animal proteins, may be linked to a reduced risk of CKD [16]. Similar to our study, other studies also looked at the link between plant and animal protein and the risk of MetS and other cardiometabolic diseases [53–55]. Azmati et al. [53] found that higher animal protein consumption and the ratio of animal protein to plant protein are associated with cardiometabolic risk factors such as increased WC and FPG. Their study revealed that including a substantial amount of plant protein in the total protein intake has positive effects on cardiometabolic risk factors. Previous studies have mainly compared the effects of plant and animal protein [53–56], but there is limited research on a comprehensive protein quality score including total protein and the ratio of plant to animal protein. This study is unique and examines these relationships for the first time.

Another important and researchable aspect of diet in the etiology of MetS is the effects related to the length of the food supply chain. The length of the food supply chain can play a crucial role in predicting the onset and development of MetS due to the possible impact they have on the quantity and quality of the nutrients in the foods [57, 58]. Previous investigations reported that a long supply chain (LSC) that refers to a complex network of intermediaries involved in the movement of food products from the point of origin to the point of consumption may be associated with a higher risk of MetS [57, 58]. Because in this condition, an unfavorable impact on the quality and quantity of nutrient content of food items may occur. However, short supply chains (SSCs), are more direct pathways for food products from producers to consumers with fewer intermediaries involved in transportation or storage processes. SSCs can result in fresher products due to shorter transportation times. Therefore, in SSCs, the quantity and quality of nutrients such as proteins and CHOs are optimally maintained and an SSCs-based diet can be associated with reducing the risk of MetS [57, 58]. It should be noted that a limitation of the current study is that we did not have data on the supply chain (LSC and SSC) of the food consumed by the participants. Considering the effect that this factor may have on the quality of the nutrient content of foods, having such information could be useful in a better evaluation of the relationships observed in this study as well as a more comprehensive conclusion.

Some limitations of the current study should be mentioned. First, using questionnaires for estimating data for physical activity and dietary intake can cause measurement errors and recall bias, but to minimize the errors we used valid and reliable questionnaires which were specially developed for the Iranian population. Second, we used the USDA FCT to assess nutrient and energy intake from participants’ diets due to incomplete Iranian FCT. Third, since alcoholic drinks such as wine and beer are not common or maybe unreported in the Iranian population due to religious considerations and legal restrictions; therefore we could not determine individuals’ information for alcohol consumption, which could have played a confounding factor in the analysis of the current study. Another limitation of the current study is that dietary data was assessed solely at baseline, which may not capture potential changes in food patterns and nutrient intake that could occur during a 6-year follow-up, however, research in nutritional epidemiology has suggested that eating habits and dietary patterns in individuals does not find significant changes at least in 5–10 years. Finally, even after adjusting for various confounding factors, residual confounding from unknown or unmeasured variables like genetic background or psychological stress cannot be ignored. However, the strengths of the present study should also be mentioned. It is the first large population-based study to investigate the cause-effect relationship between a CQI and protein scores diet and the risk of MetS. The follow-up period (over 6 years) was suitable for identifying the notable occurrence of MetS. Dietary patterns and physical activity data were collected using valid and reliable questionnaires.

## Conclusions

Our results indicated that a diet with a higher CQI and protein score was significantly associated with a reduced risk of MetS in adults. Also, some of their components showed a linear relationship with the 6-year changes of MetS components. The findings of the present study can greatly contribute to a better understanding of the impact of the quality of macronutrients, regardless of their quantity, on the risk of chronic diseases, especially MetS. These findings can be confirmed by more future prospective studies that will examine the relationship between the dietary quality of protein and CHOs and the risk of MetS and its components.

### Electronic supplementary material

Below is the link to the electronic supplementary material.

Supplementary Material 1

## Data Availability

The datasets analyzed in the current study are available from the corresponding author on reasonable request.
