# Supplementary figures and images for: Macronutrients quality indices and risk of metabolic syndrome and its components in Iranian adults
Source: BMC Cardiovasc Disord. 2024 Feb 26;24:126. doi: 10.1186/s12872-024-03779-1 (PMC10898212; doi:10.1186/s12872-024-03779-1)

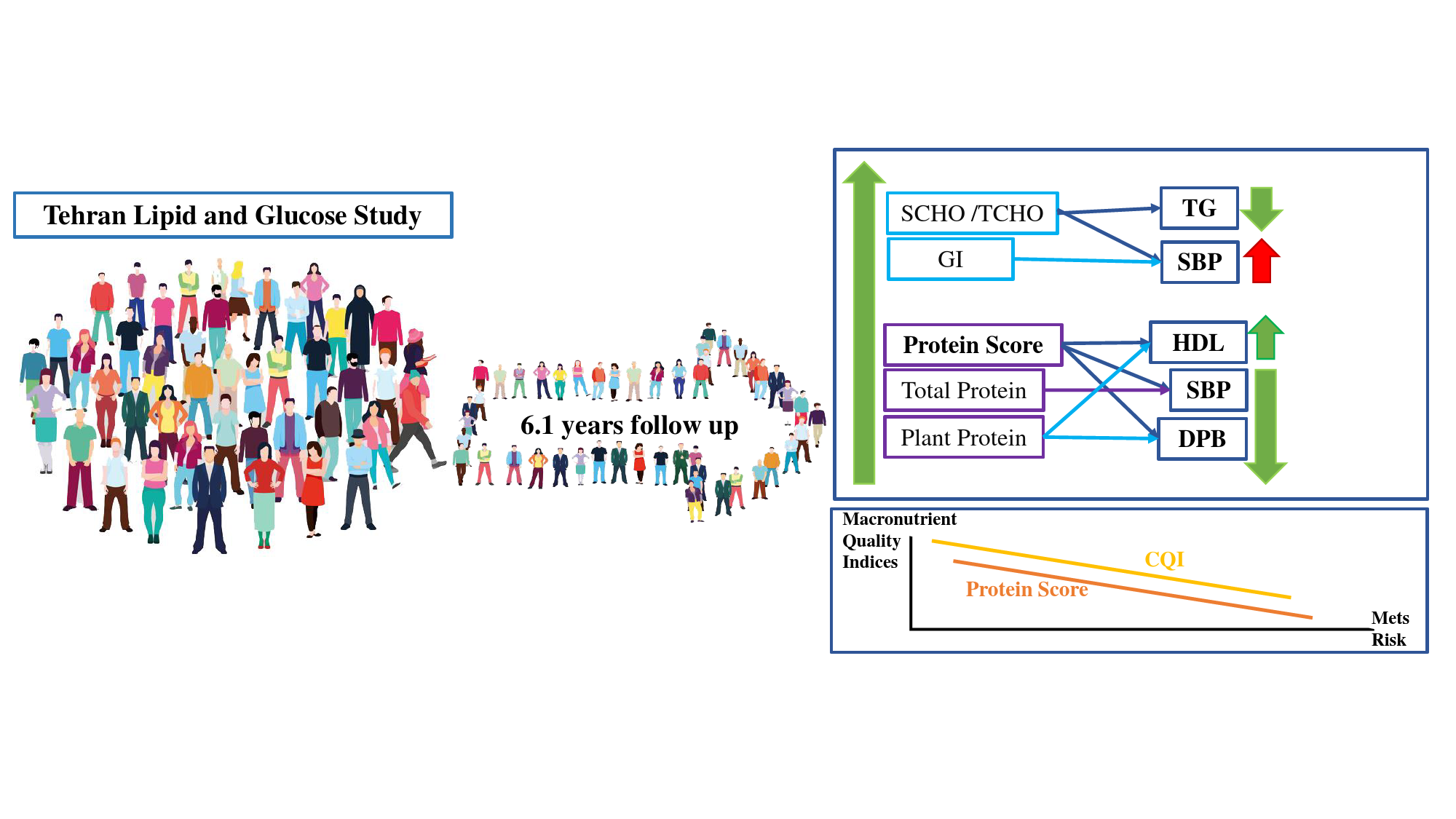

Supplement: Supplementary file 1 — Supplementary Material 1 [file 12872_2024_3779_MOESM1_ESM.tiff]
